# Evaluation of Risk Factors and Injury Mechanisms for Soft Tissue Knee Injuries

**DOI:** 10.7759/cureus.89509

**Published:** 2025-08-06

**Authors:** Thomas Molloy, Benjamin D Gompels, Simone Castagno, Matthew Dowsett, Andrew McCaskie, Stephen McDonnell

**Affiliations:** 1 Department of Trauma and Orthopaedics, University of Cambridge, Cambridge, GBR; 2 Department of Surgery, University of Cambridge, Cambridge, GBR; 3 Department of Trauma and Orthopaedics, Addenbrooke's Hospital, Cambridge University Hospitals NHS Foundation Trust, Cambridge, GBR; 4 Department of Trauma and Orthopaedics, Cambridge University Hospitals NHS Foundation Trust, Cambridge, GBR

**Keywords:** knee joint soft tissue, ligamentous knee injury, meniscus tear, predictive risk factors, risk factors

## Abstract

Identifying risk factors is essential in diagnosing and preventing soft tissue knee injuries (STKIs). These risk factors are broadly categorised into patient (intrinsic) and external (extrinsic), and non-modifiable and modifiable. Non-modifiable factors predispose individuals to injury, while modifiable ones offer opportunities for intervention and prevention. Injury mechanisms are also important for biomechanical consideration and can help guide diagnosis. Literature on risk factors and mechanisms is limited by its focus on specific subgroups, such as elite athletes or single injury types, reducing generalisability. This study aimed to identify potential associations of patient factors, external factors, and injury mechanisms in patients presenting with acute knee injuries. Eighty-five patients were recruited in a major regional Urgent Treatment Centre over 18 months and completed a digital questionnaire capturing relevant risk factors and injury details. Male sex was the only risk factor significantly associated with an increased risk of injury (RR 1.51, 95% CI 1.05-2.0), while hyperextension was the only injury mechanism significantly linked to injury (RR 2.90, 95% CI 1.2-6.4). This study highlights the inherent difficulties of diagnosing acute STKIs, stemming from nonspecific symptoms and variably reported injury mechanisms. Future research should integrate biomechanical assessments with clinical and contextual risk factors to improve diagnostic accuracy and develop targeted prevention strategies for high-risk athletic populations.

## Introduction

Recognising and understanding risk factors are critical in diagnosing and treating soft tissue knee injuries (STKIs). These factors, which encompass a range of variables from individual characteristics to lifestyle choices, have a significant impact on the occurrence, severity, and long-term consequences of knee injuries. Current literature suggests a multifactorial contribution to an increased risk of injury, categorising the risk factors for STKI as either patient-related (intrinsic) or external (extrinsic). Patient factors can be further subdivided into non-modifiable and modifiable [1-3].

Non-modifiable patient factors play a significant role in predisposing individuals to STKIs and include sex, age, previous knee injury, family history of STKIs, and generalised joint hypermobility (GJH). Women have been found to exhibit a higher incidence of STKIs, potentially due to anatomical (wider Q-angle, smaller ligament size), biomechanical (greater dynamic valgus), and hormonal (increased ligament laxity via estrogen and relaxin) differences [2,4-8]. Age has also been reported as critical, with peak STKI incidence occurring in individuals under 30 due to rapid skeletal growth, insufficient neuromuscular control, and high-risk sports participation [9-12]. Previous STKIs, of which anterior cruciate ligaments (ACLs) have the highest prevalence, are shown to be strong predictors of reinjury or contralateral damage, especially in younger men returning to sport at high intensity following reconstruction [10,13-15]. A family history of STKIs has been reported to increase susceptibility through inherited biomechanical traits and behavioural patterns such as shared sports participation [16]. GJH, which is often hereditary, contributes to joint instability and may double the risk of STKIs [3,17,18].

Modifiable patient factors that influence STKI risk include body mass index (BMI), biomechanical function, and neuromuscular control. An elevated BMI increases axial loading and dynamic valgus forces on the knee, thereby heightening the risk of injury [2,13,17,19,20]. Poor biomechanics, including abnormal joint alignment or movement patterns, can lead to increased stress on soft tissue structures. Similarly, when neuromuscular control is insufficient, dynamic stability is impaired as forces are redirected from the active support of muscles and tendons to the passive restraint of ligaments. This results in increased tensile loading of ligamentous structures, making them more susceptible to injury during sudden or high-stress movements such as pivoting or landing [8,21]. Unlike intrinsic factors, these modifiable elements provide key opportunities for intervention through targeted training, injury prevention programmes, and weight management strategies.

External factors that affect STKI risk include the level of participation, type of exposure, playing surface, weather conditions, and footwear. STKIs can result from both contact and non-contact mechanisms, with non-contact injuries making up the majority, while contact injuries frequently involve more severe multi-structure knee damage [22]. Sports can be classified by high and low risk levels, with high-risk sports involving high-energy manoeuvres such as cutting, pivoting, landing from jumps, decelerating suddenly, and making sharp turns (e.g., rugby, football, skiing). Higher levels of participation, such as elite or collegiate athletes, are associated with an increased risk of injury due to greater intensity, frequency, and physical demands [23]. Similarly, STKIs have been reported to be more common and severe during competition than training [24]. Weather conditions, such as dryness or cold, increase ground friction and resistance, potentially heightening the risk of injury [3].

The mechanism of injury involves the specific traumatic forces experienced through the knee that caused the damage. The primary descriptions are whether the knee underwent hyperextension, a valgus (medially directed) or varus (laterally directed) force, or a twisting force [25]. These classical presentations of various STKIs demonstrate that specific patient history details are valuable diagnostic tools for distinguishing acute STKIs. However, it is essential to remember that these indicators have significant cross-over and will allow a clinician to establish only a general diagnostic framework.

Existing literature on risk factors for STKIs is drawn from heterogeneous populations, with many studies focusing narrowly on elite athletes, single-sport cohorts, or isolated injury types (e.g., ACL ruptures). This specificity, while useful for understanding risk within those contexts, limits the external validity of findings when applied to broader or more diverse athletic populations. Several systematic reviews and meta-analyses have highlighted the variability in injury mechanisms and risk profiles between recreational and professional athletes and between contact and non-contact sports [1,3,9,21]. These disparities underscore the challenge of generalising findings across athlete levels, sexes, and mechanisms of injury.

The objective of this study was to collect comprehensive data on both intrinsic and extrinsic risk factors, as well as mechanisms of injury, across a diverse population of individuals presenting to the Urgent Treatment Centre (UTC) with an acute knee injury. This will enable the evaluation of their predictive value for STKIs and validate their relevance in line with existing literature, informing risk screening and injury prevention programs.

## Materials and methods

This prospective observational study was conducted over 18 months from October 2023 to March 2025 in the UTC and the Trauma and Orthopaedics Clinic at Addenbrooke’s Hospital, Cambridge, United Kingdom. The study population comprises individuals aged 16 years and older who present with a primary complaint of an acute knee injury. Inclusion criteria for the study are patients aged over 16 years, presenting with an acute knee injury sustained within the last 72 hours, and attending the UTC. Patients were excluded if they were under 16 years of age, presented with a non-acute knee injury (i.e., more than 72 hours since injury), or had confirmed bony injuries. Additional exclusion criteria include the presence of neurovascular compromise, suspected knee dislocations, clinical intoxication at the time of presentation, multiple injuries, or if the patient is considered vulnerable due to the traumatic nature of the injury, such as cases requiring safeguarding or psychosocial intervention.

A total of 85 patients presenting with acute knee injuries at the time of their index presentation were recruited. At the time of initial presentation, patients completed a digital questionnaire that captured a range of patient-related factors and external factors. Details of the mechanism of injury were also recorded (Appendices). Clinical management proceeded according to standard pathways, and those involved in clinical decision-making were blinded to the risk factor data to avoid influencing treatment decisions. Patient outcomes were analysed prospectively using electronic medical records. The outcome of “soft tissue knee injury” was defined as the MRI-confirmed diagnosis of a grade two or three ligamentous injury or any meniscal injury. These data points were collected systematically to assess their association with the risk of soft tissue knee injury.

Continuous variables were assessed using the median and interquartile range (IQR). For categorical variables, relative risks were calculated, and p-values were determined using Fisher's exact test. The p-value tested the null hypothesis that the risk ratio equalled 1, with the alternative hypothesis being that the risk ratio was not equal to 1. A p-value of <0.05 was considered statistically significant. Multiple logistic regression and Fisher’s exact tests were performed using GraphPad Prism version 10.1.0 for MacOS (GraphPad Software, Boston, Massachusetts, USA; www.graphpad.com).

There was missing data for two variables: age (n = 2) and BMI (n = 10). No imputation was performed for these missing variables. All other questionnaire items were fully completed by all participants. To ensure diagnostic accuracy, there was a delay between the end of data collection and the start of analysis, allowing sufficient time for clinical management pathways to be commenced and diagnoses to be confirmed.

This study was approved by the Cambridge University Hospitals NHS Foundation Trust Research and Development Department and received ethical approval from the Health Research Authority (HRA) and the Northern Ireland Research Ethics Committee (REC). The study was conducted under IRAS ID: 327031 and REC Reference: 23/NI/0136. All participants provided informed consent before their inclusion. Data was stored securely on REDCAP Safe Haven.

## Results

Among the 85 patients, 52 (61%) were male. Within the injured group, 15 out of 18 patients (83%) were male. The overall median age for patients (n = 83) was 32 years (interquartile range (IQR): 25-45). Injured patients (n = 18) had a median age of 29.5 years (IQR: 19.75-32.25), whereas non-injured patients (n = 65) had a median age of 33 years (IQR: 25-47). Regarding BMI, the median value for all patients (n = 75) was 24.9 (IQR: 22.5-28.2). Injured patients (n = 16) had a median BMI of 25.4 (IQR: 24.13-28.63), while the non-injured group (n = 59) had a median BMI of 24.4 (IQR: 21.7-28.2). There were two missing values for age and 10 missing values for BMI. There were no significant differences in age or BMI between the injured and non-injured groups (Table 1).

**Table 1:** Patient Demographics for 85 Patients Presenting to the Urgent Treatment Centre with Acute Soft Tissue Knee Injuries There were two missing values for age and 10 missing values for BMI.

Demographic	Overall	Injured	Non-Injured
Sex (Male)	52 (61%) (n=85)	15 (83%) (n=18)	37 (55%) (n=67)
Age (years, Median, IQR)	32 (25–45) (n=83)	29.5 (19.75–32.25) (n=18)	33 (25–47) (n=65)
BMI (kg/m^2^, Median, IQR)	24.9 (22.5–28.2) (n=75)	25.4 (24.13–28.63) (n=16)	24.4 (21.7–28.2) (n=59)

**Figure 1:**
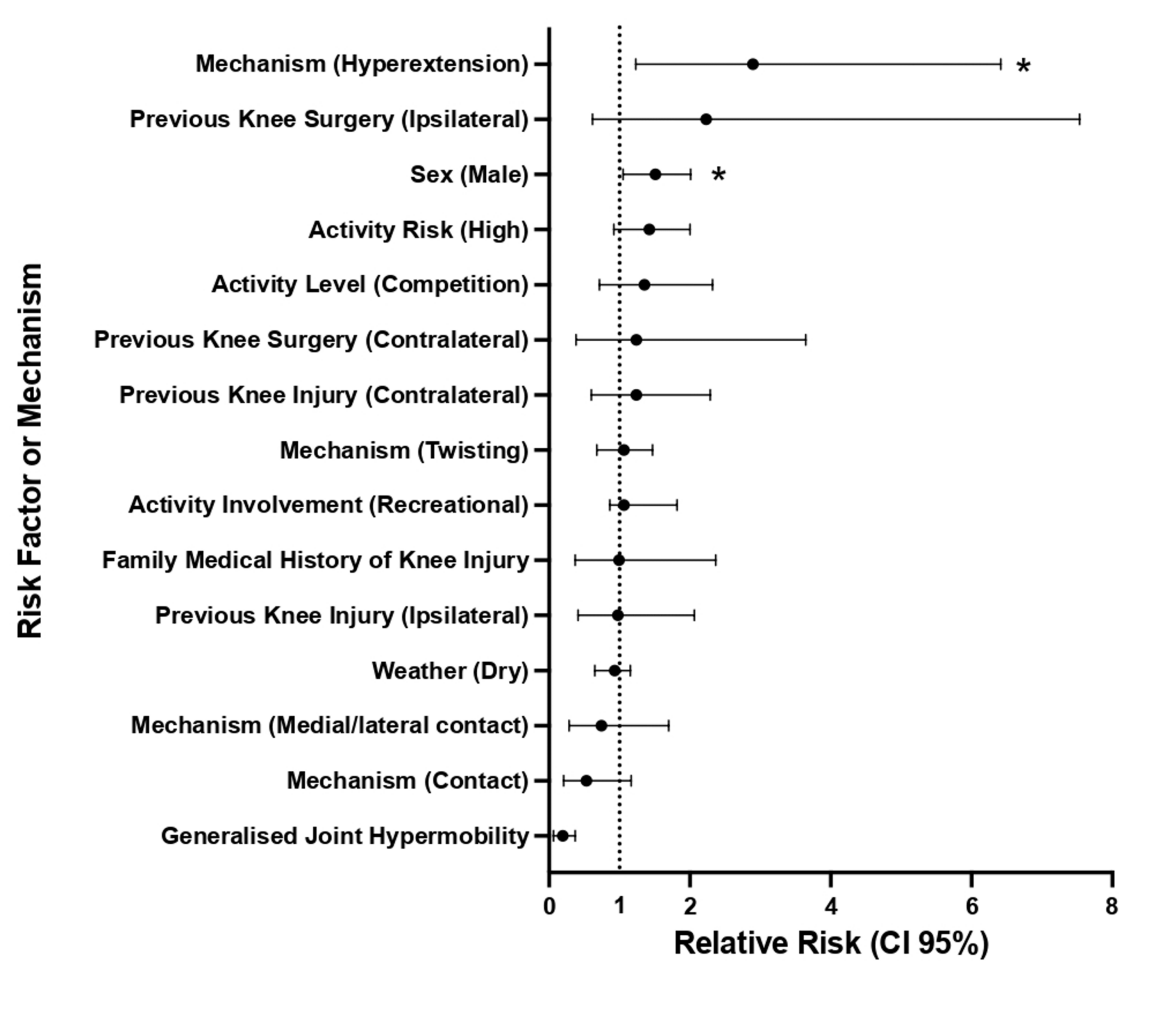
Dot and Whisker Plot of Relative Risks (95% Confidence Intervals) for Risk Factors or Mechanisms of Soft Tissue Knee Injury Statistical significance of p<0.05 is defined by (*) for sex and hyperextension.

Patient factors assessed included generalised joint hypermobility (RR 0.37, 95% CI 0.06-0.19, p=0.444), previous ipsilateral knee injury (RR 0.98, 95% CI 0.41-2.1, p>0.999), previous contralateral knee injury (RR 1.24, 95% CI 0.60-2.3, p=0.580), previous contralateral knee surgery (RR 1.24, 95% CI 0.38-3.6, p=0.712), previous ipsilateral knee surgery (RR 2.23, 95% CI 0.62-7.5, p=0.357), family history of knee injury (RR 0.99, 95% CI 0.37-2.4, p>0.999), and sex (male) (RR 1.51, 95% CI 1.1-2.0, p=0.033). Of these factors, only male sex demonstrated a statistically significant association with injury risk (Table 2, Figure 1).

**Table 2:** Relative Risks, 95% Confidence Intervals, p-Values for Risk Factors and Injury Mechanisms of Soft Tissue Knee Injuries Statistical significance of p<0.05 is defined by (*) for sex and hyperextension.

Risk Factor or Mechanism	Relative Risk (significance)	95% Confidence Interval	p-value
Patient Factors			
Family Medical History of Knee Injury	0.99	0.37 – 2.4	>0.999
Generalised Joint Hypermobility	0.37	0.06 – 0.19	0.444
Previous Knee Injury (Contralateral)	1.2	0.60 – 2.3	0.58
Previous Knee Injury (Ipsilateral)	0.98	0.41 – 2.1	>0.999
Previous Knee Surgery (Contralateral)	1.2	0.38 – 3.6	0.712
Previous Knee Surgery (Ipsilateral)	2.2	0.62 – 7.5	0.357
Sex (Male)	1.5 (*)	1.1 – 2.0	0.033
External Factors			
Activity Involvement (Recreational)	1.1	0.86 – 1.8	0.568
Activity Level (Competition)	1.4	0.72 – 2.3	0.514
Activity Risk (High)	1.4	0.92 – 2.0	0.118
Weather (Dry)	0.93	0.65 – 1.2	0.728
Mechanism			
Contact	0.53	0.21 – 1.2	0.174
Medial/lateral contact	0.74	0.28 – 1.7	0.769
Twisting	1.1	0.68 – 1.5	>0.999
Hyperextension	2.9 (*)	1.2 – 6.4	0.034

External factors included dry weather (vs. slippery/wet) (RR 0.93, 95% CI 0.65-1.2, p=0.728), recreational activity (vs. professional activity) involvement (RR 1.06, 95% CI 0.86-1.8, p=0.568), competition level activity (vs. training level) (RR 1.35, 95% CI 0.72-2.3, p=0.514), and high-risk activity (vs. low-risk activity) (RR 1.42, 95% CI 0.92-2.0, p=0.118). None of these external factors reached statistical significance (Table 2, Figure 1).

Injury mechanisms assessed were contact (vs non-contact) (RR 0.53, 95% CI 0.21-1.2, p=0.174), medial/lateral contact (vs non-contact) (RR 0.74, 95% CI 0.28-1.70, p=0.769), twisting (vs no twisting) (RR 1.06, 95% CI 0.68-1.5, p>0.999), and hyperextension (vs no hyperextension) (RR 2.90, 95% CI 1.2-6.4, p>0.036). Only hyperextension was significantly associated with an increased risk of injury (Table 2, Figure 1).

## Discussion

Soft tissue knee injuries are a common problem among individuals participating in various activities [24]. The causes of these injuries have been reported to be multifactorial, attributed to both patient- and external-risk factors, as well as injury mechanisms. Identifying these risk factors and mechanisms has been critical in developing injury prevention strategies for STKIs [25].

For patient factors, our findings reported that only male sex was a significant risk factor for predicting STKIs. We noted that men are 1.5 times more likely to have sustained an injury than women in individuals presenting to UTC with acute knee injuries. This may be due to the significant behavioural impact of men’s increased rates of sporting participation, particularly their involvement in high-risk sports and risk-taking behaviour [26-28]. It is important to note that sex discrepancies reported in the literature state that women are up to eight times more likely to sustain an injury when compared at equal playing time in all-sex sports [6]. An interpretation of this is that while the proposed sex influences of anatomical and neuromuscular variations remain as important considerations for women involved in sports, men may still have an overall higher incidence of injury [12].

For external factors, no statistically significant associations with STKIs in this cohort were observed. However, the point estimates for high-risk and competitive activity suggest a possible trend toward increased injury risk that may warrant further investigation in larger populations. 

Four injury mechanisms were evaluated for their relationship to STKIs, reflecting a range of biomechanical scenarios commonly encountered in athletic or traumatic settings. Hyperextension was the only mechanism that demonstrated a statistically significant association with STKIs. This suggests that hyperextension may be a uniquely damaging and specific mechanism affecting various soft tissue structures of the knee, leading to a markedly increased risk of injury. This mechanism involving excessive anterior translation or posterior capsule strain could explain its stronger predictive value [24]. Twisting mechanisms and medial/lateral contact showed no statistically significant associations. While twisting is frequently implicated in ACL and meniscal injuries, its lack of specificity may reduce its predictive value when assessed broadly across all STKIs. General contact injuries were associated with a reduced relative risk, although this association was not statistically significant. This might reflect the varied presentations of contact injuries, which may involve multiple trauma patterns and complicate their isolated analysis [24,25]. Overall, injury mechanisms are inherently non-specific, encompassing a broad range of forces and situations. Among them, hyperextension stands out as the most specific and strongly associated mechanism with STKIs in this cohort, potentially serving as a valuable focus for clinical suspicion and injury prevention strategies.

Despite a wide range of variables assessed, most individual risk factors and mechanisms did not reach statistical significance in predicting STKIs. This lack of isolated predictive power underscores the complexity and heterogeneity of injury presentations, where no single factor is sufficient to reliably indicate pathology. These findings emphasise the need for diagnostic clustering, in which combinations of patient characteristics, external factors, and injury mechanisms are analysed together to improve diagnostic precision. Such an approach better reflects the multifactorial nature of STKIs and supports the development of clinically useful risk stratification tools.

Limitations of this study include the modest sample size and it being conducted at a single centre, which limit the generalisability of the findings to broader populations or other clinical settings. As such, any trends should be interpreted cautiously, given the exploratory nature of the study. A larger, multicentre study is critical to validate these results and assess the predictive value of risk factors across diverse cohorts. Furthermore, it is essential to note that biomechanical and neuromuscular factors, such as dynamic valgus collapse, proprioception, and motor control deficits, were not assessed in this study; however, they are known to play a crucial role in injury risk and may further explain the lack of significance observed with individual clinical factors [29]. However, a key strength lies in the prospective, patient-recorded, and standardised collection of all variables, allowing for consistent data capture at the point of presentation.

## Conclusions

In conclusion, this study highlights the inherent difficulty of diagnosing STKIs in the acute setting, where symptoms are often nonspecific, and injury mechanisms are variable and inconsistently reported. Future research should focus on integrating biomechanical assessments with clinical and contextual risk factors to improve diagnostic accuracy and develop targeted prevention programmes, particularly for high-risk populations in sport and physical activity.

## Appendices

**Table 3:** Participant Questionnaire Provided to Patients on Initial Presentation in the Urgent Treatment Center. The Questionnaire Was Filled Out Electronically and Recorded in REDCap

Question	Answer Options	
The following questions refer to your past medical history:		
What is your sex?	Male/Female/Other	
What is your date of birth?	Day/Month/Year	
What is your weight?	kg/stone	
What is your height?	cm/inches	
Have you ever experienced a past injury to the soft tissue structures of the knee that you have currently injured? This includes ligaments, meniscus, or cartilage.	Yes/No/Unsure	
Have you ever experienced a previous injury to the soft tissue structures of the other knee?	Yes/No/Unsure	
Have you had past surgery on your injured knee?	Yes/No/Unsure	
Have you ever had past surgery on your other knee?	Yes/No/Unsure	
Do you have increased joint flexibility? For example, do any of the following apply to you: Have you been referred to as “double-jointed”? Do you consider yourself to have increased flexibility? Do you easily dislocate your joints? Do your joints “pop out”? Do your elbows extend beyond the normal angle? Can you touch your thumb to your wrists?	Yes/No/Unsure	
Has anyone in your immediate family previously had a significant soft tissue injury to their knee? This includes ligaments, meniscus, or cartilage.	Yes/No/Unsure	
The following questions refer to the circumstances and situation under which your injury occurred:		
Was the activity you were participating in during your injury considered high risk or low risk? The following are considered high risk: Basketball, soccer, lacrosse, rugby, American football, handball, field hockey, skiing, volleyball and squash. The following are considered low risk: Ice hockey, tennis, baseball, cross-country running, Golf, cycling, jogging and weight training. If your activity is not listed, please choose the category of an activity most similar to your activity.	High Risk /Low Risk	
What sport or activity were you participating in at the time of injury?	[Free text]	
What was your level of participation?	Recreational/Professional/Other	
What type of activity were you doing at the time of injury?	Training/Competition/Other	
What type of surface were you on when injured?	Grass/Artificial Turf/Hard Floor/Other	
What type of footwear were you wearing at the time of injury?	Studded Boots/Trainers/Barefoot /Other	
The following questions refer to experiences you may have felt during your injury:		
During your injury, what level of pain did you experience significant pain in your knee? 0 is no pain, and 10 is the worst pain you could possibly imagine.	1–10 scale	
During the injury, did you experience any twisting or pivoting of your knee during the injury?	Yes/No/Unsure	
During the injury, did your knee hyperextend during the injury? Hyperextension is where the knee is straightened beyond its normal angle or limit.	Yes/No/Unsure	
During the injury, did you experience direct impact to the inside or outside of your knee?	Yes/No/Unsure	
During the injury, did you hear or feel a “popping”, “cracking”, or “tearing” sensation in your knee?	Yes/No/Unsure	
The following questions refer to experiences from after your injury:		
In the hours after your injury, could you weight bear on your injured knee? Weight-bearing is defined as either: Being able to continue with your activity or being able to take 4 steps unsupported (two on each leg), regardless of any limping.	Yes /No/Unsure	
In the hours after your injury, was your ability to complete your day-to-day activities impacted?	Yes/No/Unsure	
In the hours after your injury, did you notice significant swelling of the injured knee?	Yes/No/Unsure	
Did you notice if the swelling was rapid or delayed? Rapid swelling appears within hours. Delayed swelling appears within days.	Rapid/Delayed	
Did you notice significant bruising on the injured knee?	Yes/No/Unsure	
The following questions refer to experiences since your injury:		
Since your injury, have you lost a significant amount of movement in your injured knee?	Yes/No/Unsure	
Since your injury, have you experienced any “locking”, “catching”, or “clicking” in your injured knee?	Yes/No/Unsure	
Since your injury, have you experienced instability, “giving way”, or “shifting” in your injured knee?	Yes/No/Unsure	
This is the end of the questionnaire. Thank you for your time.		
